# Serum lipoprotein(a) and reclassification of coronary heart disease risk; application of prediction in a cross-sectional analysis of an ongoing Iranian cohort

**DOI:** 10.1186/s12889-023-17332-w

**Published:** 2023-12-02

**Authors:** Mojgan Ghavami, Alireza Abdshah, Sadaf Esteghamati, Nima Hafezi-Nejad, Manouchehr Nakhjavani, Alireza Esteghamati

**Affiliations:** 1 Cardiovascular research institute, Tehran Heart Center, Tehran University of Medical Sciences, Tehran, Iran; 2 Endocrinology and Metabolism Research Center (EMRC), Vali-Asr Hospital, School of Medicine, Tehran University of Medical Sciences, P.O. Box 13145-784, Tehran, Iran; 3 Division of Biostatistics, Department of Public Health Sciences, University of Miami Miller School of Medicine, Miami, FL USA; 4 School of Medicine, Tehran University of Medical Sciences, Tehran, Iran; 5 Department of Radiology, Johns Hopkins Hospital, Baltimore, MD USA

**Keywords:** Lipoprotein(a), Metabolic syndrome, Coronary Heart Disease, Novel biomarker, Integrated discrimination index, Net reclassification improvement, Framingham Risk score

## Abstract

**Introduction:**

Recent studies have introduced elevated lipoprotein(a) (Lp(a)) as a risk factor for coronary heart disease (CHD). This study investigated whether the addition of Lp(a) as a novel biomarker to the Framingham Risk Score (FRS) model improves CHD risk prediction.

**Methods:**

The study included 1101 Iranian subjects (443 non-diabetic and 658 diabetic patients) who were followed for 10 years (2003–2013). Lp(a) levels and CHD events were recorded for each participant.

**Results:**

The Net Reclassification Index (NRI) after adding Lp(a) to the FRS model was 19.57% and the discrimination slope was improved (0.160 vs. 0.173). The Akaike Information Criterion (AIC), a measure of model complexity, decreased significantly after adding Lp(a) to the FRS model (691.9 vs. 685.4, *P* value: 0.007).

**Conclusions:**

The study concluded that adding Lp(a) to the FRS model improves CHD risk prediction in an Iranian population without making the model too complex. This could help clinicians to better identify individuals who are at risk of developing CHD and to implement appropriate preventive measures.

## Introduction

Coronary heart disease (CHD) is the leading cause of death worldwide and a major risk factor for disability-adjusted life years, especially among young people [1, 2].

Hypertension, dyslipidemia, tobacco smoking, diabetes, physical inactivity, and diet are known as some conventional risk factors for CHD [3]. Recently, several novel biomarkers such as C-reactive protein, B-type natriuretic peptide, lipoprotein(a) (Lp(a)), and homocysteine have been introduced as stronger predictors for CHD risk assessment [4].

Framingham Risk Score (FRS) which is one of the scoring systems used for the evaluation and prediction of CHD in 10 years, categorizes patients as “high”, “intermediate,” and “low” risk. FRS variables include some conventional risk factors such as age, diabetes, smoking, hypertension, total cholesterol, high-density lipoprotein cholesterol (HDL-C), and low-density lipoprotein cholesterol (LDL-C) [5–8]. Despite utilizing this scoring model, many people who were actually in the high-risk group were not detected or were categorized as low-risk. Meanwhile, many people who are categorized as high-risk according to this model are at intermediate risk. Thus, decision-making in these groups is troubling. Previous studies have shown that novel biomarkers are the most useful factors in predicting patients with an intermediate risk of coronary heart disease [9, 10].

There are already studies regarding the adaptation of the Framingham model for cardiovascular disorder in different populations, showing that the model can be adjusted slightly for different populations to better accommodate the differences [11, 12]. Also recently, new methods have been introduced to increase the precision of previous coronary heart disease prediction models by utilizing novel biomarkers [13, 14].

Lp(a) is an LDL-like particle that contains a hydrophilic, highly glycosylated protein called apolipoprotein(a) bound to apolipoprotein B100 by a disulfide bridge. Lp(a) plays a significant role in atherosclerosis as an independent risk factor for cardiovascular diseases [15, 16].

Recent studies suggested that Lp(a) levels were independently associated with an increased risk of coronary artery disease, even after accounting for traditional risk factors such as LDL-C levels. Incorporating Lp(a) measurement into risk stratification models significantly could improve risk prediction and potentially lead to identifying individuals who may benefit from more aggressive personalized preventive strategies [17, 18].

A statistical measure used to evaluate the performance of predictive models is the area under the curve (AUC) with the range of 0.5 (no discrimination) to 1.0 (perfect discrimination) which represents the overall discriminatory ability of a model in distinguishing between individuals with and without the outcome of interest (CHD event in this study). The net reclassification index (NRI) and integrated discrimination index (IDI) are another statistical measure that assesses the improvement in risk prediction when adding a new marker to an existing model. NRI takes into account the correct reclassification of individuals into higher or lower-risk categories, as well as incorrect reclassification. It calculates the net proportion of individuals who are correctly reclassified into a higher risk category minus the net proportion of individuals who are incorrectly reclassified into a lower risk category with the range from − 2 to + 2. IDI quantifies the difference in average predicted risks between individuals with and without events and ranges from − 1 to + 1. Positive values indicate improved prediction with the addition of the new marker. NRI and IDI can be used alongside the AUC to further evaluate and compare different models in predicting CHD [19–21].

This study aims to investigate whether adding Lp(a) to the FRS model improves its predictive value, as measured by NRI and IDI.

## Materials and methods

### Study population

In this retrospective cohort study, the target population was selected from the patients who were referred to the endocrinology and metabolism clinic of Vali Asr Hospital in Tehran, Iran, between 2003 and 2013. We included all the patients who underwent coronary angiography for highly suspected CHD. The patients with missing data on lipid profile and also the subjects with diabetes mellitus who were Insulin-dependent, type 1, secondary, or pancreatitis-related with a prior history of other endocrine diseases (except their diabetes status) were excluded. The diabetic patients included in the study were on oral antidiabetic agents.

Informed consent was obtained from each patient and the study was reviewed and approved by the board of medical ethics at Tehran University of Medical Sciences with the code of IR.TUMS.REC.1394.412.

The diabetes was diagnosed according to the American Diabetes Association criteria [22].

Finally, 1101 patients were enrolled in this study (two sub-groups: 443 non-diabetic and 658 diabetic patients).

### Demographics, examinations, laboratory tests, and other health parameters

Age, gender, weight (kg), height (cm), waist circumference (cm), systolic and diastolic blood pressure (SBP and DBP, respectively) (mmHg), and medical history of the patients were recorded.

Waist circumference (WC) is defined as the midline between the lowest rib and the iliac crest.

SBP and DBP were measured from both arms after 15 min of rest in the supine position and the mean value was recorded. Venous blood samples were collected after 12 h of overnight fasting.

To assess fasting plasma glucose (FPG) and 2-hour post-prandial plasma glucose (2hPP), a glucose oxidase assay was used. Lipid profiles including total cholesterol, HDL-C, LDL-C, and triglyceride (TG) were measured using a direct enzymatic method (Parsazmun, Karaj, Iran).

To measure fasting insulin, radioimmunoassay using separate specific antibodies was utilized (Immunotech, Prague, Czech Republic). Serum creatinine was assessed by the Jaffe method. HbA1c was evaluated by high-performance liquid chromatography (HPLC, DS5 Pink kit; Drew, Marseille, France). Also, Lp(a) was measured by turbidometry method (Binding site, SPA plus, Birmingham, United Kingdom).

Body Mass Index (BMI – Kg/m^2^) was calculated as Weight/Height^2^ and Homeostasis Model Assessment of Insulin Resistance (HOMA-IR) as [FPG (mg/dL) ×Fasting Insulin(U/L)] / 405. Metabolic syndrome was determined according to the nationally modified version of the International Diabetes Federation (Modified- IDF) criteria [23]. Self-reported cigarette smoking in the preceding year was recorded for analysis.

### Statistical analysis

Baseline characteristics in patients who had developed CHD were compared to the group who had not developed CHD, by T-test and Chi-square for continuous and categorical variables, respectively.

To calculate the FRS predictions for the 10-year risk of CHD, FRS sheets were used [8]. A generalized linear model based on the binomial family was used to compare the prediction vs. occurrence of events in conventional FRS and FRS plus Lp(a), separately. For each model, the likelihood ratio Chi-square, Akaike Information Criterion (AIC), Bayesian information criterion (BIC), and AUC of the receiver operating characteristic (ROC) curve were reported. We also reported the incremental change in the AUC of ROC and NRI [24].

AIC and BIC describe model complexity. The discrimination power of these two models was evaluated by the AUC of ROC. To determine the value of the additive marker, C-statistics, and the AUC are not efficient enough. Therefore, novel methods such as IDI and NRI were conducted in this study [24].

The data analysis was performed by R using packages base [25], tidyverse [26], pROC [27], lmtest [28], and perdictABEL [29]. A two-sided *P* < 0.05 was considered significant.

## Results

Our study included 1101 subjects and patients who developed CHD had significantly higher levels of FPG, HOMA- IR, and SBP. They also had lower levels of LDL-C and total cholesterol. Additionally, they were more current smokers and older. CHD was more common in males. Also, we observed a significantly higher proportion of patients with metabolic syndrome who had developed CHD. The summary of patients’ characteristics is listed in Table 1. We have also investigated the adjusted association between Lp(a) and metabolic syndrome and observed that each 10-unit increase in Lp(a) is associated with 7% odds of having metabolic syndrome. The details of the unadjusted and adjusted odds ratios of having metabolic syndrome per 1- and 10-unit increase in Lp(a), using multivariable logistic regression, are listed in Table 2.

**Table 1:** Patients’ characteristics:

	Unit (or categories)	Without CHD (966) Mean (SD) N (%)	With CHD (135) Mean (SD) N (%)	Total (1101) Mean (SD) N (%)	*P* Value
Age	Years	50.8 (11.4)	60.8 (8.14)	52.0 (11.5)	< 0.001 T-test
Sex	Female	606 (62.7%)	48 (35.6%)	654 (59.5%)	< 0.001 Chi-square
	Male	359 (37.3%)	87 (64.4%)	446 (40.5%)	
Body Mass Index	(kg/m^2^)	29.4 (5.02)	29.5 (5.00)	29.4 (5.02)	0.7 T-test
Diabetes	No	428 (44.3%)	15 (11.1%)	443 (40.2%)	< 0.001 T-test
	Yes	538 (55.7%)	120 (88.9%)	658 (59.8%)	
Fasting Plasma Glucose	mg/dL	128 (46.1)	155 (56.1)	131 (48.3)	< 0.001 T-test
Insulin	U/mL	10.4 (6.99)	10.2 (5.63)	10.4 (6.84)	0.8 T-test
HOMA-IR		3.25 (2.42)	3.76 (2.22)	3.32 (2.40)	0.01 T-test
Post prandial glucose	mg/dL	189 (86.7)	226 (88.7)	195 (88.0)	< 0.001 T test
HbA1C	%	6.71 (1.64)	7.58 (1.75)	6.82 (1.68)	< 0.001 T-test
Triglyceride	mg/dL	171 (98.5)	165 (85.2)	170 (96.9)	0.5 T test
High-Density Lipoprotein Cholesterol	mg/dL	48.1 (13.6)	44.9 (11.2)	47.7 (13.4)	0.003 T-test
Total Cholesterol	mg/dL	195 (41.6)	176 (49.1)	193 (43.0)	< 0.001 T-test
Low-Density Lipoprotein Cholesterol	mg/dL	114 (33.4)	98.4 (40.2)	112 (34.6)	< 0.001 T-test
Systolic Blood Pressure	mmHg	122 (14.8)	131 (17.5)	123 (15.4)	< 0.001 T-test
Diastolic Blood Pressure	mmHg	79.4 (7.78)	79.6 (10.1)	79.4 (8.11)	0.8 T-test
History of Hypertension	No	687 (71.1%)	50 (37.0%)	737 (66.9%)	< 0.001 Chi-square
	Yes	278 (28.8%)	85 (63.0%)	363 (33.0%)	
Creatinine	mg/dL	0.970 (0.176)	1.05 (0.185)	0.980 (0.179)	< 0.001 T-test
Metabolic syndrome	No	403 (41.7%)	29 (21.5%)	432 (39.2%)	< 0.001 Chi-square
	Yes	556 (57.6%)	104 (77.0%)	660 (59.9%)	
Smoking (%)	No	770 (79.7%)	99 (73.3%)	869 (78.9%)	0.1 Chi-square
	Yes	196 (20.3%)	36 (26.7%)	232 (21.1%)	
Lipoprotein a	<=10 mg/dL	239 (24.7%)	25 (18.5%)	264 (24.0%)	0.1 Chi-square
	> 10 mg/dL	727 (75.3%)	110 (81.5%)	837 (76.0%)	
Lipoprotein a	<=30 mg/dL	659 (68.2%)	78 (57.8%)	737 (66.9%)	0.02 Chi-square
	> 30 mg/dL	307 (31.8%)	57 (42.2%)	364 (33.1%)	
Lipoprotein a	<=50 mg/dL	803 (83.1%)	91 (67.4%)	894 (81.2%)	< 0.001 Chi-square
	> 50 mg/dL	163 (16.9%)	44 (32.6%)	207 (18.8%)	

**Table 2:** Incidence and Global Goodness of Fit estimates of Metabolic Syndrome and Metabolic Syndrome components for prediction of coronary heart disease

	Lipoprotein (a) (per 1 unit increase)		Lipoprotein (a) (per 10 units increase)	
	Odds Ratio	Increased risk (%)	Odds Ratio	Increased risk (%)
Block 1	1.007 (1.004–1.011)***	0.7%	1.076 (1.035–1.119)***	7.6%
Block 2	1.007 (1.004–1.011)**	0.7%	1.072 (1.026–1.120)**	7.2%
Block 3	1.008 (1.003–1.012)**	0.8%	1.078 (1.029–1.130)**	7.8%
Block 4	1.007 (1.003–1.012)**	0.7%	1.076 (1.028–1.128)**	7.6%

Values are Odds Ratios (95% confidence interval) from binary logistic regression analysis for Lipoprotein (a) as a continuous variable

Block 1: Unadjusted

Block 2: Age- and sex-adjusted

Block 3: Additional adjustment for low-density lipoprotein cholesterol, systolic blood pressure, and high-density lipoprotein cholesterol

Block 4: Additional adjustment for diabetes and smoking status

*P < 0.05, **P < 0.01, ***P < 0.001

**Fig. 1:**
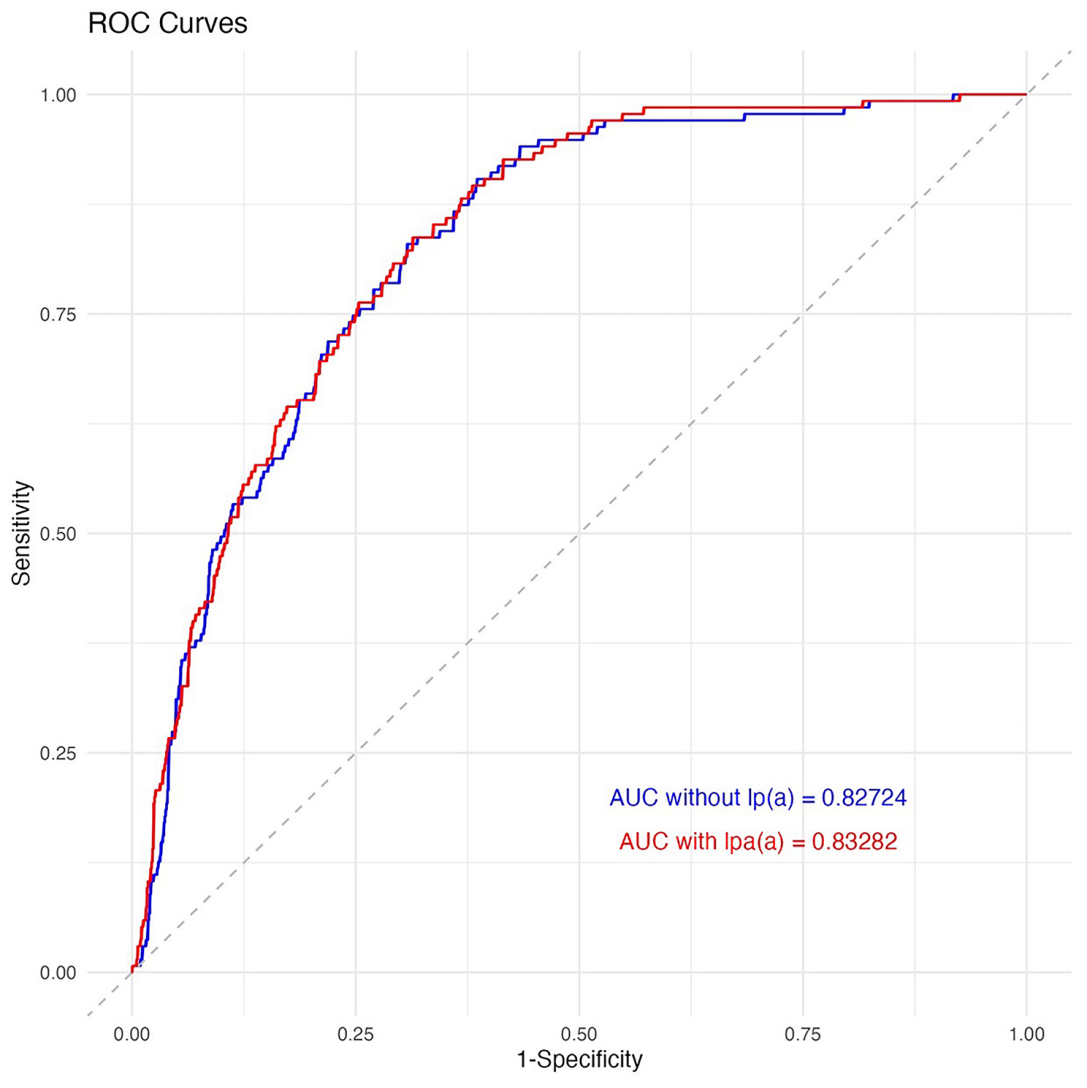
Receiver Operating Characteristic curves with and without Lipoprotein (a) with their corresponding Area Under Curves ROC: Receiver Operating Characteristic AUC: Area Under Curve Lp(a): Lipoprotein (a)

We used two models to predict the risk of CHD in our subjects: the conventional FRS model and the FRS model with Lp(a) added. We found that the model with Lp(a) had a higher likelihood ratio (Chi-square 6.53, *P* value = 0.011), improved AIC, BIC, and − 2 log-likelihood, and a small improvement in the AUC of the ROC curve. While the increase in AUC was not statistically significant (*P* = 0.200), it is important to note that it is a step towards improving the model. We expect that the addition of more novel biomarkers, one by one, will lead to a significant improvement in the model through these small increases for each biomarker. We also observed increases in discrimination slope and NRI. The details of the models are listed in Table 3. The ROC curves for both models, along with their corresponding AUCs, are shown in Fig. 1.

**Table 3:** Model characteristics of each model:

Model details	Full model (with Lipoprotein (a))	Reduced model (the original Framingham)	Difference and Test
Likelihood Ratio Chi-square	136 (*P* < 0.001)	130 (*P* < 0.001)	Chi-Square = 6.53 *P* = 0.011
AIC	685.5	692	
BIC	695.5	702.1	
-2 Log Likelihood	5593	5605	
AUC of receiver operating characteristic	0.833 (0.796–0.863)	0.827 (0.794–0.859	0.006 (*P* = 0.200, DeLong test)
Discrimination slope	0.173	0.160	IDI = 0.014 IDI = 8.75%
NRI			19.57%

**AIC**: Akaike Information Criterion; Measures of model complexity, weighting the additional information of a model against its entropy; a lower value indicates an increased global fit

**BIC**: Bayesian information criterion: It is a criterion for model selection, closely associated with AIC. BIC, like AIC, is penalty-based measure, with increased penalty (compared to AIC) for the increasing number of parameters; a lower value indicated a better model fit

**AUC**: Area Under the Curve using receiver operating characteristic curve statistics; Measures of discrimination; a higher value indicates better discrimination of events vs. non-events

**IDI**: Integrated Discriminant Improvement; Measures the difference between the discrimination slopes of two models before and after the addition of Lipoprotein (a). Discrimination slope in the binary context is defined as the difference between mean predicted probabilities of events and non-events; a higher value indicates a larger improvement

**NRI**: Net Reclassification Improvement; Measuring the percent of reclassified subjects, among events and non-events; a higher value indicates better implication of the added marker

## Discussion

We assessed the risk of CHD occurrence in 1101 subjects, including 443 non-diabetic and 658 diabetic patients, over 10 years using a new risk assessment model (FRS plus Lp(a)). Our study showed that adding Lp(a) to a model based on conventional risk factors improved NRI and IDI in the Iranian population. It also decreased the model complexity.

Unexpectedly, patients with CHD had lower TG, LDL-C, and total cholesterol levels. This may be due to treatment effects, lifestyle modifications, or early detection and treatment of secondary causes of dyslipidemia, such as hypothyroidism, during CHD management.

A previous study of 826 subjects aged 45 to 84 years in Bruneck, Italy, found that elevated Lp(a) predicts 15-year cardiovascular disease (CVD) outcomes, and that adding Lp(a) to the FRS model improves CVD risk prediction. The study showed that the NRI related to Lp(a) was 17.1% and 22.5% for patients with and without CVD, respectively, and 39.6% overall [14].

Another study on 8720 Danish participants over 17 years which was in line with our study showed that the addition of extreme levels of Lp(a) to the baseline model improved CHD event classification. In other words, after adding Lp(a) to the conventional model at the levels of ≥ 80th (47 mg/dl) and ≥ 95th (115 mg/dl) percentiles, 23% and 39% of patients with myocardial infarction (MI) and 12% and 25% of patients with CHD were correctly reclassified. For these two cut points, the NRI was 16% and 23% for MI and 3% and 6% for CHD, respectively. However, Lp(a) addition over the entire concentration did not significantly change the NRI. Discrimination was improved by IDIs for MI and CHD in extreme levels of Lp(a), while C-index changes remained insignificant [30].

A study that assessed three cohorts of women including the Women’s Health Study (WHS, N = 24,558), Women’s Health Initiative Observational Study (WHI, N = 1815), and the Justification for Use of Statins in Prevention (JUPITER) trial (N = 2,569) found that increased Lp(a) is associated with higher CVD risk only among patients with high total cholesterol (above 220 mg/dL). According to the mentioned study, prediction improvement among women also was minimal. In contrast, JUPITER showed a strong association of Lp(a) with CVD among men with low total cholesterol levels [31].

A recent retrospective study that enrolled 1395 patients, found better capability of an existing model for predicting coronary artery disease after adding Lp(a) compared to other lipid parameters. The new model which incorporated Lp(a) achieved an NRI of 12.8% and significant improvement in accuracy, measured by IDI [32].

One of the strengths of our study is that it was conducted in a Middle Eastern population and there are not many studies in this population on this issue. However, there are certain limitations. The study was done on retrospective data. Most of the patients with CHD were on Statin and/or other anti-lipid agents which may affect the level of Lp(a) and other lipid parameters. We only included one novel biomarker in this study. The study lacked data on the details of the major adverse cardiac events. For future studies, we will also continue enrolling patients to assess the continued effects of Lp(a) and other novel biomarkers, in a cohort with an increased sample size and increased number of events, to further improve the statistical power of our study as well.

## Conclusion

This study showed that adding Lp(a) to the FRS model compared to the conventional FRS is superior in risk stratification, discrimination, and net reclassification in a sample of the Iranian population. For future studies, we’d recommend prospective designs with a larger sample size including more novel biomarkers, in hopes of better establishing predictive models for our population.

## Data Availability

The data is part of an ongoing cohort. Deidentified data and R codes can be made available upon reasonable request from the corresponding author.
